# Effects of C/N ratio on the growth and protein accumulation of heterotrophic *Chlorella* in broken rice hydrolysate

**DOI:** 10.1186/s13068-022-02204-z

**Published:** 2022-10-08

**Authors:** Yihui Cai, Ligong Zhai, Xiaoman Fang, Kangping Wu, Yuhuan Liu, Xian Cui, Yunpu Wang, Zhigang Yu, Roger Ruan, Tongying Liu, Qi Zhang

**Affiliations:** 1 State Key Laboratory of Food Science and Technology, Nanchang University, Nanchang, 330047 Jiangxi China; 2 College of Food Engineering, Anhui Science and Technology University, Fengyang, 233100 Anhui China; 3 China Coal Zhejiang Testing Technology Co, Ltd., Hangzhou, 310000 China; 4 Australian Centre for Water and Environmental Biotechnology (Formerly AWMC), The University of Queensland, St. Lucia, Brisbane, QLD4072 Australia; 5 Center for Biorefining and Department of Bioproducts and Biosystems Engineering, University of Minnesota, 1390 Eckles Ave., St. Paul MN, 55108 USA; 6 Jiangxi Maternal and Child Health Hospital, Nanchang, 330006 Jiangxi China

**Keywords:** Heterotrophic, *Chlorella vulgaris*, Protein, Broken rice hydrolysate, Amino acid

## Abstract

**Background:**

Microalgae protein is considered as a sustainable alternative to animal protein in the future. Using waste for microalgal culture can upgrade low-value raw materials into high-value products, helping to offset the cost of microalgal protein production. In this study we explored the feasibility of using microalgae heterotrophic fermentation to convert broken rice hydrolysate (BRH) into protein.

**Results:**

The results showed that the increase of BRH supplemental ratio was beneficial to the increase of biomass production but not beneficial to the increase of intracellular protein content. To further improve protein production, the effect of C/N ratio on intracellular protein accumulation was studied. It was found that low C/N ratio was beneficial to the synthesis of glutamate in microalgae cells, which in turn promoted the anabolism of other amino acids and further the protein. When the C/N ratio was 12:1, the biomass productivity and protein content could reach a higher level, which were 0.90 g/L/day and 61.56%, respectively. The obtained *Chlorella vulgaris* biomass was rich in essential amino acids (41.80%), the essential amino acid index was as high as 89.07, and the lysine content could reach up to 4.05 g/100 g.

**Conclusions:**

This study provides a theoretical basis and guidance for using *Chlorella vulgaris* as an industrial fermentation platform to convert broken rice into products with high nutritional value.

## Background

Over the next 30 years, the global demand for food will continue to rise as a result of continued population and consumption growth [1]. Global meat demand is expected to double by the middle of this century, making the search for new food sources a goal of the utmost importance [2]. To address the threat of climate change and environmental pollution while ensuring food security for the world's population, reducing meat consumption or replacing them with more sustainable sources of protein is unavoidable [3, 4]. Microalgae, as a potential source of non-animal protein, has the advantages of fast growth, short growth cycle, high protein content and no occupation of cultivated land [5, 6]. In addition, this makes them a promising new source of protein to meet the nutritional needs of the world's population, as well as reducing pressure on the environment [7, 8]. *Chlorella vulgaris* (*C. vulgaris*) is an edible single-cell microalgae strain that has been commercially produced for more than 50 years [9]. In recent years, the evolution of *C. vulgaris* production in the world was remarkable [10, 11]. However, it is still very small compared to the huge nutritional requirements. It is urgently needed to improve biomass production and protein content as well as reduce production costs.

*Chlorella vulgaris* could be cultured in heterotrophic, mixotrophic and autotrophic modes [12, 13]. Comparing to the other two culture modes, heterotrophic culture mode present: (i) higher growth and production rates; (ii) does not require light; (iii) can be cultured in sealed fermenter to avoid contamination by the environment; (iv) can achieve higher biomass concentration, thus simplifying the harvesting process and reducing the cost of harvesting [14, 15]. However, heterotrophic cultivation requires glucose as an organic carbon source, which increases the cost of *C. vulgaris* production. To achieve large-scale commercialization of microalgae heterotrophic culture, it is necessary to develop cheap alternative carbon sources for industrial application of heterotrophic culture [16]. In this regard, cheap and abundant starch by-products produced by food processing industry are good candidates [17].

Broken rice is a by-product of rice processing, and its starch content is up to 75% [18]. As a major rice processing country, China’s broken rice produced during rice processing is conservatively estimated to be 30 million tons per year [19]. Because of its high starch content and abundant annual yield, broken rice has become a good alternative raw material for ethanol [20], pigment [21] and rice wine [22] by industrial fermentation. This indicates that it is economically feasible to use microbial fermentation to convert broken rice into high value-added products. However, using broken rice hydrolysate for the heterotrophic culture of *C. vulgaris* to obtain plant protein has not been well-reported. The main component in the hydrolysate of broken rice is glucose, in addition to which there are many other unknown soluble substances, such as oligosaccharides and soluble proteins. The effects of these substances on the biomass and protein accumulation of heterotrophic *C. vulgaris* remain unclear.

The aim of this research was to heterotrophic culture *C. vulgaris* to produce protein using broken rice hydrolysates as carbon source, and to improve the protein content of heterotrophic microalgae by adjusting the amount of carbon and nitrogen sources in the media. The biomass production, nutrients utilization, cell morphology and chemical composition of *C. vulgaris* which heterotrophic cultured under different addition amounts of broken rice hydrolysate and initial C/N ratio were analyzed. The nutritional value of heterotrophic *C. vulgaris* was also evaluated by amino acid pattern analysis and compared with soybean protein. This study provides an approach for the high value-added utilization of broken rice resources and provides guidance for the production of high protein heterotrophic *C. vulgaris*.

## Results and discussion

### Effects of BRH supplemental ratio on the growth of *C. vulgaris*

**Fig. 1:**
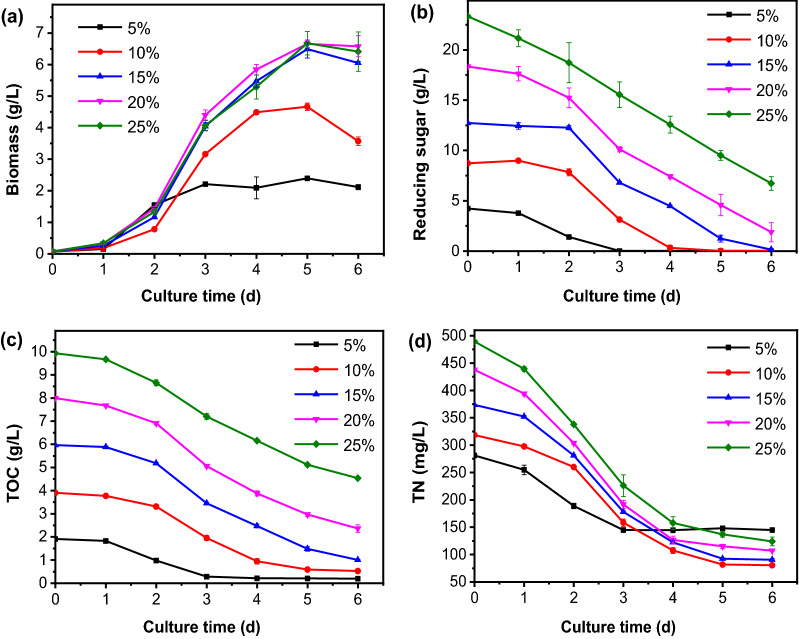
Effects of BRH supplemental ratio on the growth of *C. vulgaris*. **a** Biomass production; (**b**) Reducing sugar concentration of culture media; (**c**) TOC concentration of culture media; (**d**) TN concentration of culture media. Error bars represent standard deviation from the mean of three replicates

As shown in Fig. 1a, the effects of BRH supplemental ratios on the growth of *C. vulgaris* were illustrated. When BRH supplemental ratio increased from 5% to 15%, biomass productivity increased significantly from 0.34 to 1.00 g/L/day (*P* < 0.05). When BRH supplemental ratio increased from 15% to 25%, there was no significant difference in biomass productivity, which all exceeded 1.00 g/L/day. This indicated that BRH addition ratio was no longer a key factor affecting algae growth when it exceeded 15%.

The consumption of reducing sugars in the media is shown in Fig. 1b. The initial concentration of reducing sugar in the media were different due to the different proportion of BRH. With the extension of incubation time, the concentration of reducing sugar in the medium showed a decreasing trend. When the BRH supplemental ratio was 5%, 10% and 15%, the reducing sugar concentration in the media was reduced to 0 g/L at the 3rd, 4th and 5th day, respectively. When the BRH supplemental ratio was 20% and 25%, the reducing sugars in the medium were not completely consumed at the end of culture (in the 6th day). The decreasing trend of total organic carbon (TOC) concentration in the media was the same as that of reducing sugar (Fig. 1c). This indicted that, when BRH supplemental ratio was lower than 15%, the carbon source and energy required by heterotrophic metabolism of microalgae were insufficient, thus limiting the growth of microalgae. When the BRH supplemental ratio was larger than 15%, the organic carbon source in the medium could provide more carbon source and energy for the growth of *C. vulgaris*, thus achieving a higher biomass productivity.

The consumption of total nitrogen (TN) in the media is shown in Fig. 1d. In addition to carbon source, BRH also contains 0.81 g/L TN, which may be derived from the soluble nitrogen compounds in broken rice. Therefore, the higher the proportion of BRH, the higher the initial concentration of TN. With the extension of incubation time, the concentration of TN in the medium decreased gradually and then tended to be stable. In addition, TN was not completely consumed at the end of culture. This is because some nitrogen-containing organic matter in BRH cannot be metabolized by *C. vulgaris*.

Protein content and productivity is shown in Table 1. When the proportion of BRH was 5%, the protein content of microalgae was the highest (58.51%). These results indicated that low BRH supplemental ratio was beneficial to intracellular protein accumulation. TC/TN (mass/mass) increased from 6.79 to 20.31 when BRH supplemental ratio increased from 5% to 25%. This indicated that, the increase of BRH supplemental ratio would lead to the increase of C/N ratio in the medium, resulting in the appearance of a relatively deficient nitrogen content, thus inhibiting the protein anabolism in cells. Low C/N ratio (low BRH supplemental ratio) was not conducive to biomass production (Fig. 1a), and high C/N ratio (high BRH supplemental ratio) was not conducive to the increase of protein content (Table 1). To increase protein productivity, new strategies need to be developed to increase both biomass productivity and protein content. Therefore, the effects of C/N ratio on *C. vulgaris* biomass and protein accumulation need to be further explored. In addition, to avoid the waste of carbon source, a BRH supplemental ratio of 15% was selected for further study.

**Table 1:** Consumption of nutrients in the media and the protein content of microalgae

	Biomass productivity (g/L/day)	Reducing sugar consumption rate (%)	TOC consumption rate (%)	TN consumption rate (%)	Protein content (%)	Protein productivity(g/L/day)	Growth yield (g biomass/g TOC)
BRH supplemental amount							
5%	0.34 ± 0.01^c^	100 ± 0.00^a^	90.02 ± 0.06^a^	48.53 ± 0.38^d^	58.51 ± 0.01^a^	0.20 ± 0.01^c^	1.23 ± 0.05^a^
10%	0.58 ± 0.02^b^	100 ± 0.00^a^	86.61 ± 0.03^ab^	74.76 ± 0.99^b^	56.25 ± 0.50^b^	0.33 ± 0.01^b^	1.06 ± 0.05^b^
15%	1.00 ± 0.01^a^	98.86 ± 0.00^ab^	87.46 ± 0.30^b^	79.36 ± 0.52^a^	40.64 ± 0.01^d^	0.40 ± 0.01^a^	1.22 ± 0.01^a^
20%	1.08 ± 0.06^a^	87.75 ± 5.17^b^	76.22 ± 1.64^c^	78.10 ± 0.27^ab^	38.41 ± 0.15^e^	0.42 ± 0.03^a^	1.17 ± 0.07^ab^
25%	1.06 ± 0.10^a^	71.19 ± 2.93^c^	23.91 ± 0.56^d^	66.81 ± 2.11^c^	42.55 ± 0.50^c^	0.45 ± 0.06^a^	1.19 ± 0.14^ab^
C/N ratio							
5:1	0.94 ± 0.02^bc^	100 ± 0.00^a^	81.53 ± 0.19^b^	14.20 ± 2.29^f^	58.26 ± 0.14^c^	0.55 ± 0.01^a^	1.28 ± 0.04^ab^
7:1	0.96 ± 0.02^ab^	100 ± 0.00^a^	84.25 ± 1.03^a^	40.21 ± 0.77^e^	57.31 ± 0.06^d^	0.55 ± 0.01^a^	1.29 ± 0.02^ab^
9:1	0.93 ± 0.02^b,c^	100 ± 0.00^a^	84.12 ± 0.28^a^	56.78 ± 0.51^c^	59.71 ± 0.11^b^	0.56 ± 0.01^a^	1.22 ± 0.03^ab^
12:1	0.90 ± 0.02^bc^	100 ± 0.00^a^	83.74 ± 0.15^a^	74.20 ± 0.28^a^	61.56 ± 0.23^a^	0.54 ± 0.02^ab^	1.19 ± 0.02^b^
19:1	1.00 ± 0.03^a^	100 ± 0.00^a^	84.04 ± 0.42^a^	77.87 ± 0.88^a^	38.73 ± 0.13^e^	0.39 ± 0.01^c^	1.29 ± 0.06^ab^
23:1	0.89 ± 0.02^c^	88.27 ± 0.01^b^	76.23 ± 0.39^c^	74.84 ± 0.80^a^	32.29 ± 0.31^f^	0.29 ± 0.01^d^	1.25 ± 0.04^ab^
32:1	0.68 ± 0.02^d^	62.29 ± 0.75^c^	55.75 ± 0.39^d^	67.93 ± 1.57^b^	27.61 ± 0.36^ g^	0.19 ± 0.01^e^	1.32 ± 0.06^a^
48:1	0.24 ± 0.01^e^	23.69 ± 0.20^d^	23.86 ± 0.64^e^	52.15 ± 1.40^d^	37.92 ± 0.47^e^	0.07 ± 0.01^f^	1.09 ± 0.04^c^

Results are expressed as mean ± standard deviations (n = 3). Different lowercase superscript letters in the same column represent statistical difference (*P* < 0.05) on a particular property

### Effect of C/N ratio on the growth of *C. vulgaris*

**Fig. 2:**
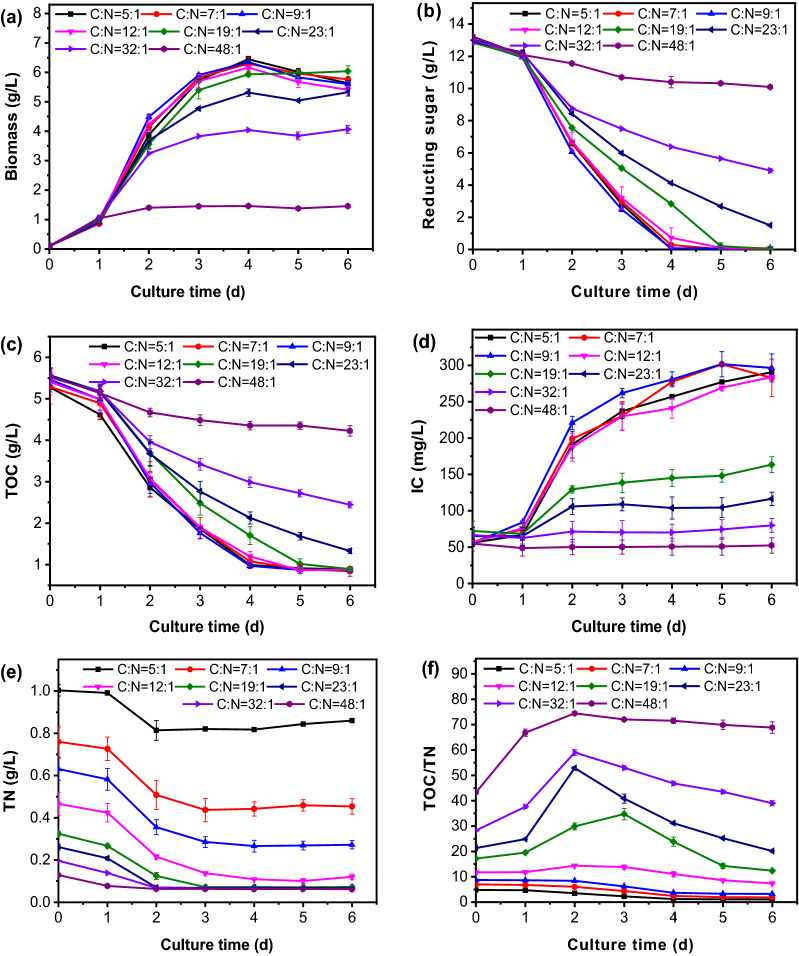
Effects of C/N ratio on the growth of *C. vulgaris* and nutrient components of media. **a** Biomass production; (**b**) Reducing sugar consumption in media; (**c**) TOC consumption in media; (**d**) Changes of IC concentration in media; (**e**) Changes of TN concentration in media; (**f**) Changes of TOC/TN in media. Error bars represent standard deviation from the mean of three replicates

The BRH supplemental ratio were kept at 15%, and the initial C/N ratio were controlled at 5:1, 7:1, 9:1, 12:1, 19:1, 23:1, 32:1 and 48:1, respectively. The biomass growth curves of *C. vulgaris* are shown in Fig. 2a. Lower C/N ratio was more beneficial to the accumulation of *C. vulgaris* biomass. When C/N ratio was less than 19:1, *C. vulgaris* grew better and biomass productivities were all more than 0.90 ± 0.02 g/L/day. The changes of reducing sugar in the media are shown in Fig. 2b. When C/N ratio was lower than 19:1, the reducing sugar consumption rate in the medium was faster. This indicated that, microalgae could intake more glucose from the medium for metabolism under the condition of sufficient nitrogen source, resulting in faster biomass growth. The consumption of TOC in the culture medium also demonstrated this phenomenon (Fig. 2c).

The concentration change of inorganic carbon (IC) in the media is shown in Fig. 2d. The lower the C/N ratio, the faster the IC released in the medium. The increase of IC in the media were mainly due to the dissolving of CO_2_ from the respiration of *C. vulgaris*. The higher the cell concentration and respiration efficiency of *C. vulgaris*, the faster the rate of CO_2_ production will be. This again proved that under the conditions of C/N ratio less than 19:1, *C. vulgaris* heterotrophic metabolism was active, leading to higher biomass production.

When the C/N ratio was less than 19:1, the TN consumption rate decreased from 77.87 ± 0.88% to 14.20 ± 2.29% (Table 1). This indicates that when C/N ratio is lower than 19:1, the amount of nitrogen source exceeds the growth and metabolism requirements of *C. vulgaris*, and the conversion efficiency of carbon source determines the biomass productivity of microalgae. However, when the C/N ratio exceeded 19:1, nitrogen source became to the limiting factor for biomass accumulation. The change of TN content in the media in Fig. 2e could well support such discussion. At the end of culture, the concentration of residual TN in the media decreased with the increase of the C/N ratio. However, when the C/N ratio exceeded 19:1, the concentration of TN in the medium was the same (65.39 ± 3.01 mg/L). In addition, TN concentration stopped decreasing on the third day when the C/N ratio was 19:1, while TN consumption in the medium stopped on the second day when the C/N ratio exceeded 19:1. This indicated that microalgae cells were in a nitrogen starvation state due to the lack of available nitrogen elements, which further affected the increase of biomass production.

The change of TOC/TN ratio in the media is shown in Fig. 2f. When the initial C/N ratio was greater than 19:1, the TOC/TN ratio in the media increased rapidly in the first 2 days and decreased after reaching the peak value. When the initial C/N ratio was less than 9:1, the TOC/TN in the medium tended to decrease with the extension of culture time. When the initial C/N ratio was 12:1, the TOC/TN ratio of the medium maintained a stable state, indicating that the consumption of carbon and nitrogen sources by *C. vulgaris* is relatively balanced.

**Fig. 3:**
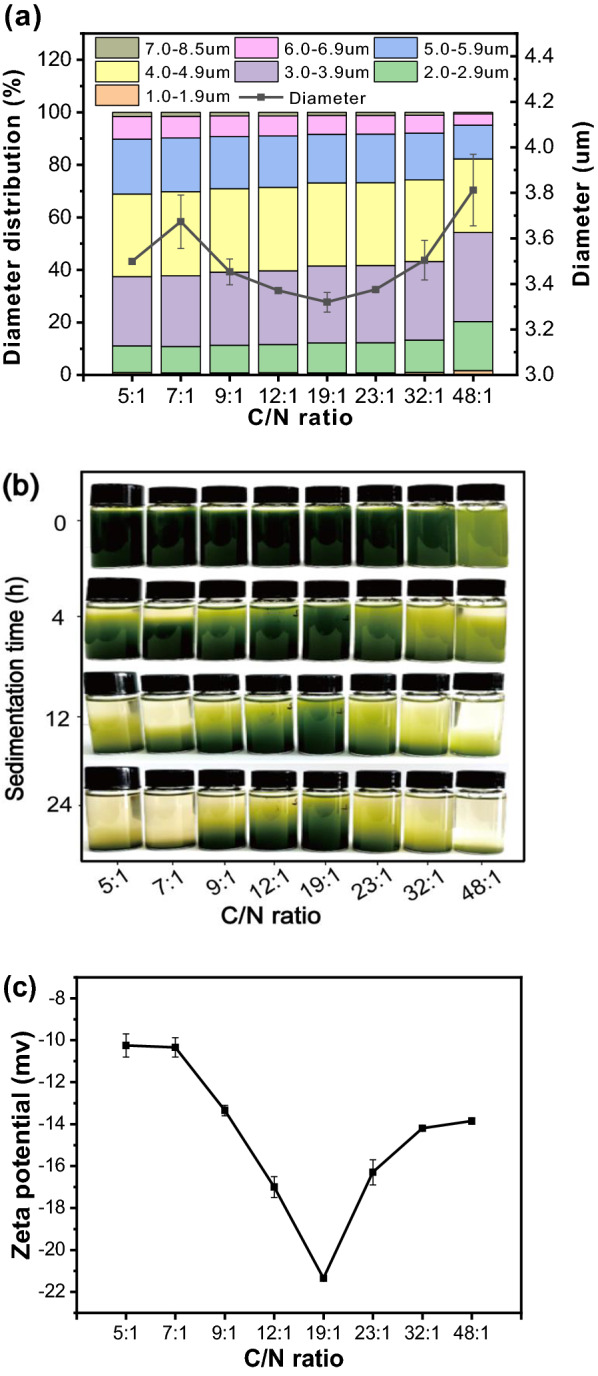
Effects of C/N ratio on the size and sedimentation of microalgal cells. **a** Size of microalgal cells; (**b**) Sedimentation of microalgal cells; (**c**) Zeta potential of microalgal cells. Error bars represent standard deviation from the mean of three replicates

Microalgal cell size is also pivotal for biomass productivity and intracellular metabolism. Planktonic algae growth requires the uptake of nutrients from the medium to form new cells [23]. Small cells have a large surface area to volume ratio, which facilitate rapid assimilation of nutrients [24]. Under the condition of environmental stress, the volume of microalgae cells will increase. The cell size distribution of *C. vulgaris* is shown in Fig. 3a. The diameter of *C. vulgaris* mainly ranges from 2.0 μm to 6.9 μm. When the C/N ratio was 19:1, the mean diameter of *C. vulgaris* cell was the smallest. This indicated that the medium environment was favorable for the proliferation of *C. vulgaris* cells at a C/N ratio of 19:1, which resulted in the reduction of cell volume. Too high or too low C/N ratio inhibited the cell reproduction of *C. vulgaris* and resulted in the enlargement of cell volume.

The sedimentation of *C. vulgaris* cells is shown in Fig. 3b. When the C/N ratio was 19:1, the sedimentation rate of *C. vulgaris* was the slowest. When the C/N ratio exceeds 19:1, the higher the C/N ratio, the more likely the microalgae cells are to sedimentation. However, when the C/N ratio is lower than 19:1, the lower the C/N ratio, also the more likely the microalgae cells are to sedimentation. The Zeta potential of *C. vulgaris* was analyzed, and the results are shown in Fig. 3c. *C. vulgaris* carried a negative charge in the medium, and the charge of *C. vulgaris* increased first and then decreased with the increase of the C/N ratio. When the C/N ratio was 19:1, the charge of the microalgae cells reached the maximum. This indicates that higher or lower C/N ratio than 19:1 will cause changes in the surface charge of *C. vulgaris* cells, which will make the microalgae cells easy to sedimentation. The sedimentation of *C. vulgaris* cells was related to the degree of inhibition. It also indicated that higher or lower C/N ratio than 19:1 can inhibit the metabolism of *C. vulgaris* to a certain extent.

In conclusion, under the condition of low C/N ratio, cells were conducive to intake sufficient nutrients from the medium for metabolism, thus promoting rapid cell division and biomass accumulation. Cell division requires more enzymes, which may promote nitrogen metabolism to protein synthesis, resulting in increased protein content in the cell. To prove this conclusion, we further analyzed the intracellular components of microalgae.

### Effect of C/N ratio on the composition of microalgae

**Fig. 4:**
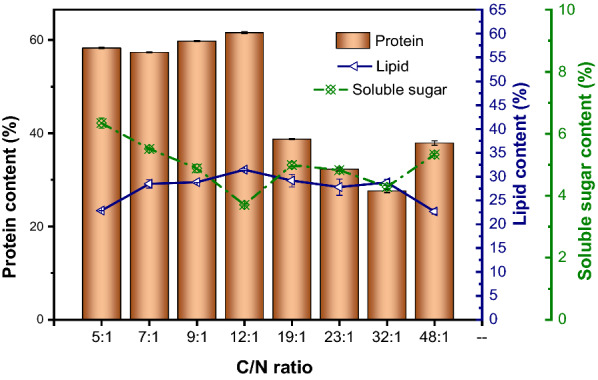
Effect of C/N ratio on the composition of microalgal cell

The effect of different C/N ratios on the protein production of *C. vulgaris* are shown in Fig. 4. When the C/N ratio was 12:1, the protein (61.56 ± 0.23%) and lipid (31.45 ± 0.17%) content in *C. vulgaris* cells were the highest, and the soluble sugar content was the lowest (3.70 ± 0.02%). The protein content of biomass was 18%, 21% and 35% higher than that of *Candida tropicalis, Pichia kudriavzevii and Saccharomyces cerevisiae, respectively* [36, 37]. This suggested that *C. vulgaris* has a higher potential for protein production than other fast-growing microorganisms. When the C/N ratio was less than 12:1, the protein content of microalgae cells had little difference and could all exceed 57.31%. When the C/N ratio increased to 19:1, the protein content decreased significantly (*P* < 0.05) with the increase of C/N ratio. These results indicated that C/N ratio had a significant effect on protein synthesis in *C. vulgaris* cells, and intracellular nitrogen was more likely to participate in protein synthesis when the C/N ratio is lower than 12:1. The influence of different C/N ratios on protein productivity is shown in Table 1. When the C/N ratio was lower than 12:1, heterotrophic *C. vulgaris* has both higher biomass productivity and higher protein content, leading to the high protein productivity (all exceeded 0.54 ± 0.02 g/L/day).

**Fig. 5:**
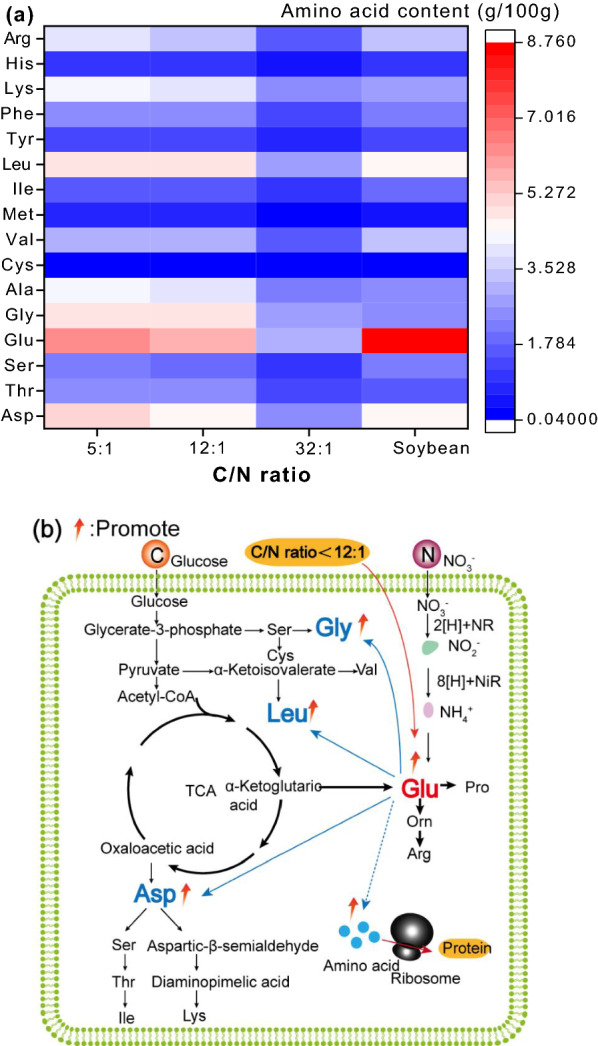
Mechanism of influence of C/N ratio on protein accumulation in *C. vulgaris* cells. **a** Changes in amino acid content; (**b**) Regulation mechanism of C/N ratio on amino acid and protein accumulation

Figure 5a shows the amino acid composition of *C. vulgaris* which heterotrophic cultured under different C/N ratios. With the decrease of C/N ratio from 32:1 to 5:1, the content of most amino acids in *C. vulgaris* cells increased significantly (*P* < 0.05). The increase of glutamic acid (Glu) was the most obvious, followed by aspartic acid (Asp), glycine (Gly) and leucine (Leu), respectively. Glu plays an important role in protein anabolism, because it is a precursor of many metabolites (Fig. 5b). In vivo, Glu is formed by reductive amination of α-ketoglutaric acid with NH_4_^+^ under the catalysis of glutamate dehydrogenase. Glutamic acid reacts with oxaloacetic acid to form ASP under the catalysis of aspartate aminotransferase [25]. Leu is produced by the reaction of α-ketoisocaproate and glutamic acid under the catalysis of leucine transferase [25]. Gly is produced by the reaction of L-serine under the catalysis of serine hydroxylmethyltransferase [25]. The synthesis of serine's precursor (3-phosphoserine) requires the involvement of glutamate. In conclusion, the reduction of C/N ratio in the medium promoted the accumulation of glutamic acid in cells, and then promoted the anabolism of other amino acids, which further contributed to the enhanced protein synthesis and accumulation.

### Nutritional profile of the protein in *C. vulgaris*

Protein is composed of amino acids, and its nutritional quality mainly depends on the content and availability of amino acids [26]. The amino acid profile of *C. vulgaris* is presented in Fig. 5a, high C/N ratio leaded to the lower protein content and further the lower amino acid content. When the C/N ratio was relatively high (32:1), the amino acid content decreased significantly (*P* < 0.05). However, under the condition of C/N ratio 12:1, large amounts of amino acids could be accumulated in *C. vulgaris* cells, and the lysine content was about 1.48 times that of soybean (Fig. 5a). Lysine is the first limiting amino acid of most grains [27–29]. If *C. vulgaris* protein is used as a nutritional supplement, it can improve the situation of insufficient dietary lysine intake. Aspartic acid and glutamic acid are the two amino acids with the highest content in *C. vulgaris*, both of which are the starting substances of lysine synthesis, so *C. vulgaris* cells contain a higher content of lysine. Amino acids such as isoleucine, leucine and valine have been listed as indispensable/essential branched chain amino acids and are recommended for the construction of muscle tissue [30]. Present study showed that the content of leucine and valine in *C. vulgaris* was higher than those in soybean.

On the basis of amino acid composition, the differences of protein nutritional characteristics between *C. vulgaris* and Soybean were evaluated (Table 2). The proportion of total essential amino acids with respect to total amino acids (E/T) of *C. vulgaris* slightly increased with the increase of C/N ratio, which was higher than that in soybean. Essential amino acid index (EAAI) is a more accurate method to evaluate the nutritional value of protein. According to previous scientific observation reports, the quality of protein foods is good when the EAAI is above 90 and is useful when EAAI ranges between 70 and 80. When the EAAI is lower than 70, the nutritional quality of food is insufficient [30]. In this study, it was found that the EAAI of *C. vulgaris* was close to 90, similar to that of soybean (EAAI 88), and there was no significant difference between the EAAI of *C. vulgaris* under different C/N ratios. This indicated that the nutritive value of *C. vulgaris* protein is comparable to that of soybean protein.

**Table 2:** Comparative nutritional profile of protein in heterotrophic *Chlorella vulgaris* and soybean

Parameter	Heterotrophic *C. vulgaris*			Soybean
	C/N = 5:1	C/N = 12:1	C/N = 32:1	
Total essential amino acids without histidine	19.61 ± 0.07^a^	19.37 ± 0.11^a^	10.92 ± 0.04^c^	17.09 ± 0.01^b^
Total non-essential amino acids	29.22 ± 0.04^a^	26.97 ± 0.11^b^	14.82 ± 0.05^c^	26.76 ± 0.06^b^
Total amino acids	48.83 ± 0.11^a^	46.34 ± 0.23^b^	25.74 ± 0.09^d^	43.85 ± 0.06^c^
E/T (%)	40.16 ± 0.05^c^	41.80 ± 0.04^b^	42.43 ± 0.01^a^	38.97 ± 0.06^d^
BV	84.87 ± 0.33^a^	85.39 ± 0.01^a^	83.29 ± 0.67^a^	84.25 ± 0.91^a^
EAAI	88.60 ± 0.31^a^	89.07 ± 0.01^a^	87.15 ± 0.61^a^	88.03 ± 0.84^a^
Total Sulphur containing amino acids	0.75 ± 0.02^a^	0.78 ± 0.01^a^	0.36 ± 0.02^b^	0.62 ± 0.05^a^
Total essential amino acids with histidine	20.60 ± 0.08^a^	20.36 ± 0.13^a^	11.46 ± 0.05^c^	18.23 ± 0.00^b^
Total neutral amino acids	28.15 ± 0.06^a^	27.68 ± 0.15^b^	15.25 ± 0.05^c^	23.38 ± 0.01^d^
Total aromatic acids	3.96 ± 0.03^a^	3.95 ± 0.03^a^	2.04 ± 0.01^c^	3.73 ± 0.00^b^
Total hydroxylic amino acids	4.61 ± 0.02^a^	4.43 ± 0.01^b^	2.58 ± 0.01^d^	4.02 ± 0.01^c^
Total acidic amino acids	11.39 ± 0.01^b^	10.29 ± 0.01^c^	5.74 ± 0.02^d^	13.34 ± 0.03^a^
Total basic amino acids	9.30 ± 0.05^a^	8.37 ± 0.07^b^	4.75 ± 0.02^d^	7.12 ± 0.02^c^
Leucine/isoleucine ratio (BCAA)	2.90 ± 0.01^a^	2.90 ± 0.01^a^	2.83 ± 0.01^b^	2.38 ± 0.01^c^
Lysine/arginine ratio	1.03 ± 0.01^c^	1.21 ± 0.01^b^	1.52 ± 0.01^a^	0.84 ± 0.00^d^

Results are expressed as mean ± standard deviations. Means in the same row with different superscripts are significantly different (*P* < 0.05)

Biological value (BV) is a measure of the percentage of the protein that is actually incorporated into human protein after digestion [30]. High quality food proteins have biological value between 70% and 100%. In this study, the biological values of *C. vulgaris* were 84.87%, 85.39% and 83.29% when the C/N ratios were 5:1, 12:1 and 32:1, respectively, indicating a high BV. There was no significant difference in BV between *C. vulgaris* protein and Soybean protein. Therefore, *C. vulgaris* can replace soybean as raw material for high protein food. *C. vulgaris* contains high concentrations of BCCA (leucine, isoleucine, and valine). They are involved in anabolic processes in protein metabolism [31]. The increase of leucine content can improve muscle protein synthesis. Thus, a diet with a higher leucine to isoleucine ratio would provide the body with more endurance. This suggests that *C. vulgaris* proteins can be used to promote muscle health. Although the unpleasant organoleptic properties of microalgae limit their use as a staple food. However, using it as a dietary supplement to develop nutrient-rich staples is crucial to improving of people's health, just like the enrichment of wheat flour with vitamins.

### Potentials of using BRH for the heterotrophic culture of *C. vulgaris*

A large amount of broken rice is produced during the process of rice processing, which causes a waste of food resources. It will bring great social and economic benefits to transform it into products with higher added value and nutritional value through microbial fermentation strategy. The present study indicated that the hydrolysate of broken rice could be used as carbon source to heterotrophic culture *C. vulgaris* for producing biomass with high nutritional value. With the development of fermentation industry, the cost of the enzyme production decreased year by year, which made its application in the production of BRH economically feasible. As shown in Table 3, the costs of BRH and glucose media were analyzed. Using broken rice hydrolysate instead of glucose can reduce the cost of media by 51.69%. In addition, the solid residue obtained in the hydrolysis process of broken rice could be used as animal feed. According to the calculation, 410.69 kg of biomass can be produced from every 1.00 t broken rice. China produces 30 million tons of broken rice every year. If it is used to heterotrophic culture *C. vulgaris*, 12.32 million tons of biomass can be produced. The produced *C. vulgaris* biomass can provide 7.584 million tons of protein, which can meet the protein requirements of 426.2 million people every year. The selling price of *C. vulgaris* biomass is 94 times that of broken rice. For every 1 t of broken rice consumed, there is a gross profit of $11,756. *C. vulgaris* biomass can be used as raw materials for health food, and can also be further extracted to obtain protein, oil and pigment. This will further increase the added value of the product. However, more studies like pilot scale operation, life cycle assessment about using BRH for the heterotrophic culture of *C. vulgaris* are needed to further confirm the feasibility and applicability of this new technology.

**Table 3:** Culture medium cost to produce 1 kg of biomass

Chemicals	Unit price^a^ (USD/kg)	Amount^b^ (g)	
		BRH group	Glucose group
NaNO_3_	0.57	243	243
K_2_HPO_4_	1.48	6.48	6.48
MgSO_4_·7H_2_O	0.07	12.15	12.15
CaCl_2_·2H_2_0	0.35	5.83	5.83
Citric acid	2.19	0.97	0.97
Ferric ammonium citrate	7.98	0.97	0.97
EDTA·2Na	4.36	0.16	0.16
Na_2_CO_3_	0.38	3.24	3.24
A_5_	1.48	73.55	97.36
α-Amylase	1.19	60.00	0
Glucoamylase	0.69	160.00	0
Glucose	0.30	0	3200
Broken rice	0.57	2370	0
Total cost^c^ (USD)		1.14	2.36

^a^The unit price is determined according to the minimum price in Wangsheng raw material trading platform in China (https://www.rawmex.cn/) in August 2022 (1.00 USD = 6.77 CNY)

^b^The amount of chemicals required to produce 1 kg of biomass

^c^Total cost is calculated based on the amount and unit price of various chemicals required to produce 1 kg of biomass

## Conclusions

The present study demonstrated that *C. vulgaris* can be effectively heterotrophic cultured with BRH medium to obtain considerable biomass and protein. The initial C/N ratio of the medium is a key regulatory factor during the heterotrophic metabolism of *C. vulgaris*. The low C/N ratio promoted the synthesis of glutamate in the cell, which further promoted the synthesis of other amino acids and protein. When the C/N ratio was 12:1, the biomass productivity, protein content and protein productivity of *C. vulgaris* were high, which were 0.90 g/L/day, 61.56%, 0.54 g/L/day, respectively. The essential amino acid content of *C. vulgaris* reached 41.80%, and its nutritional value was similar to that of soybean. The protein in *C. vulgaris* contains a higher level of lysine than cereal protein, and it is suitable as a dietary supplement to improve the lack of lysine intake in the mainly vegetarian population. This study provided a new strategy for using broken rice as fermentation material to produce high value-added products.

## Methods

### Microalgae and culture medium

The broken rice used to produce the hydrolysate in this study was the by-product of rice processing provided by Zhongkou Fu Rice Industry Co Ltd, Dongguan, China. The microalgae strain used in this study, *C. vulgaris* FACHB-32, was obtained from the Institute of Hydrobiology, Chinese Academy of Science, P. R. China. The soybeans used in this study were purchased from the market in Nanchang, China.

The preparation of hydrolysate from broken rice was based on the method former reported [18]. Briefly, broken rice was gelatinized in boiling water for 30 min, and then hydrolyzed with 0.24% α-amylase (3,700 U/g, Solarbio Science and Technology Co., Ltd.) and 0.59% glucoamylase (10^5^ U/g, Solarbio Science and Technology Co., Ltd.). After that, the sample was boiled to inactivate the enzyme, and filtered with 0.45 µm filter membrane and stored at 4 °C for later use. The concentration of TOC, reducing sugar and TN content in BRH were 39.04, 87.12 and 0.81 g/L, respectively.

### Culture conditions

Sterile microalgae strains were isolated by plate scribing method. Microalgal inoculum was cultured in BG-11 medium with 10 g/L of glucose under heterotrophic conditions. Before inoculation, the pH of all media was adjusted to 7.0 and then sterilized (121 °C, 20 min). The inoculum was centrifuged at 8000 rpm at 4 °C for 5 min, and the precipitate was washed twice with sterile deionized water. Initial inoculation concentration was controlled at (2.5 ± 0.5) × 10^6^ cell/mL. *C. vulgaris* was cultured in 500 mL conical flask with 300 mL nutrient solution in the dark. The mouth of the flask was sealed with a sterile 0.22 μm membrane. No artificial aeration was performed. The temperature was maintained at 25 °C, and the shaking speed was 120 rpm. Inoculation, sampling and culture were performed under sterile conditions. At the end of culture, microscopic examination showed that the media were free from contamination with other microorganisms.

To study the effect of BRH dosage on microalgae growth, 50 mL, 100 mL, 150 mL, 200 mL and 250 mL BRH were transferred to five volumetric flasks (1000 mL), added with nitrogen source (1.5 g sodium nitrate) and minerals (same as BG-11), and then added with deionized water to 1000 mL. Thus, the corresponding content of BRH in the media were 5%, 10%, 15%, 20% and 25% (volume of BRH/volume of the medium), respectively. Then they were sterilized and used for heterotrophic culture of microalgae to explore the effects of different BRH supplemental ratios on the growth of microalgae.

To explore the effect of C/N ratio on microalgae growth and further improve the biomass and protein production of microalgae. Under the condition of 15% BRH, the initial C/N ratio of media were controlled at 5:1, 7:1, 9:1, 12:1, 19:1, 23:1, 32:1 and 48:1 by adjusting the addition amount of sodium nitrate, respectively.

### Microalgal growth analysis

The changes in biomass production (g/L) of *C. vulgaris* in the process of the cultivation were calculated according to the method of Zhou et al. [32]. Briefly, 20 mL of *C. vulgaris* suspension was sampled and filtered with 0.22 μm filter membrane (which has been dried and weighed). Then the membrane was transferred to a vacuum drying oven and dried at 105 °C for 24 h. Biomass production was calculated according to the change of membrane weight.

The size of *C. vulgaris* cells was measured by laser particle size analyzer (Bettersize instruments LTD, BT-9300HT, China). At the end of culture, microalgae cells suspension was transferred to deionized water, and the cells were evenly dispersed by mechanical agitation, and then the cell size was measured. The refractive index values of *C. vulgaris* particles and aqueous solution were set as 1.52 and 1.33, respectively.

The zeta potential of *C. vulgaris* was measured with a zeta potential meter (Malvern, ZEN 2600, UK). At the end of culture, 1.0 mL of *C. vulgaris* cells without pH and osmotic pressure regulation were dispersed into the sample cell and inserted into the unit for measurement.

To detect the sedimentation of cells, the cell suspension was transferred into glass bottles and placed in an incubator at 25 °C. The changes of cell sedimentation were observed at 0, 4, 12 and 24 h, respectively.

### Determination of nutrient components in media

Every 24 h, 4 mL microalgae suspension was removed from the flask by sterilized pipette and transferred to a 5 mL centrifuge tube. The samples were centrifuged at 8000 rpm for 10 min under temperature of 4 °C, and then the nutrient components in the supernatant were determined. The reducing sugar content of culture media were determined by DNS method [33]. The total nitrogen (TN), inorganic carbon (IC) and total organic carbon (TOC) content of culture media were analyzed using a multi N/C 3100 analyzer (Analytik Jena AG, Jena, Germany) [34].

### Chemical composition analysis

After the culture, the microalgae sludge was separated by centrifugation at 8000 rpm for 5 min at 4 °C and washed three times with deionized water. After freeze-drying for 48 h, microalgae powder was collected to analyze the content of intracellular nutrients. Crude protein was determined by Kjeldahl analyzer (Hanon K-9860, China), and the conversion coefficient was 6.25. The total lipid content was determined by gravimetric method derived from Song et al. [35].

Soluble sugar content in *C. vulgaris* was analyzed by phenol–sulfuric acid method. The biomass was suspended with deionized water at 100 °C for 30 min, crushed with an ultrasonic cell crusher (Scientz JY96-IIN, China) for 5 min, and centrifuged at 10,000 rpm for 5 min. Then the content of soluble sugar in supernatant was analyzed.

### Amino acid composition

Amino acid composition of *Chlorella vulgaris* and soybean was determined by an amino acid analyzer (Hitachi L-8900, Japan) according to the method of Pietrysiak et al. [31]. The essential amino acid index (EAAI) and biological value (BV) were calculated according to the method of Mir et al. [30].

### Statistical analysis

The experiments were repeated three times, and the data were expressed in the form of mean ± standard deviation. The statistical significances were analyzed using one-way ANOVA (*P* < 0.05).

## Abbreviations

BRH: Broken rice hydrolysate
TOC: Total organic carbon
TN: Total nitrogen
IC: Inorganic carbon
EAAI: Essential amino acid index
*C. vulgaris*: *Chlorella vulgaris*

## References

[1] Godfray HCJ, Beddington JR, Crute IR, Haddad L, Lawrence D, Muir JF, Pretty J, Robinson S, Thomas SM, Toulmin C (2010). Food security: the challenge of feeding 9 billion people. Science.

[2] Geada P, Moreira C, Silva M, Nunes R, Madureira L, Rocha CMR, Pereira RN, Vicente AA, Teixeira JA (2021). Algal proteins: Production strategies and nutritional and functional properties. Bioresour Technol.

[3] McHardy C, Djike Kammegne T, Jänich I (2021). Energy-efficient ultrasound-assisted extraction of food proteins from the microalga *C. vulgaris* at elevated static pressure. Innov Food Sci Emerg Technol.

[4] Vogelsang-O’Dwyer M, Zannini E, Arendt EK (2021). Production of pulse protein ingredients and their application in plant-based milk alternatives. Trends Food Sci Technol.

[5] Diprat AB, Silveira Thys RC, Rodrigues E, Rech R (2020). *Chlorella sorokiniana*: A new alternative source of carotenoids and proteins for gluten-free brea*d*. Lwt Food Sci Technol.

[6] Chen R, Yang M, Li M, Zhang H, Lu H, Dou X, Feng S, Xue S, Zhu C, Chi Z, Kong F (2022). Enhanced accumulation of oil through co-expression of fatty acid and ABC transporters in *Chlamydomonas* under standard growth conditions. Biotechnol Biofuels Bioprod.

[7] Siahbalaei R, Kavoosi G, Noroozi M (2021). Protein nutritional quality, amino acid profile, anti-amylase and anti-glucosidase properties of microalgae: Inhibition and mechanisms of action through in vitro and in silico studies. Lwt Food Sci Technol.

[8] Zhang W, Li J, Zhang Z, Fan G, Ai Y, Gao Y, Pan G (2019). Comprehensive evaluation of a cost-effective method of culturing *Chlorella pyrenoidosa* with unsterilized piggery wastewater for biofuel production. Biotechnol Biofuels.

[9] Jafari Y, Sabahi H, Rahaie M (2016). Stability and loading properties of curcumin encapsulated in *Chlorella vulgaris*. Food Chem.

[10] Wiatrowski M, Klein BC, Davis RW, Quiroz-Arita C, Tan ECD, Hunt RW, Davis RE (2022). Techno-economic assessment for the production of algal fuels and value-added products: opportunities for high-protein microalgae conversion. Biotechnol Biofuels Bioprod.

[11] Sun H, Zhao W, Mao X, Li Y, Wu T, Chen F (2018). High-value biomass from microalgae production platforms: strategies and progress based on carbon metabolism and energy conversion. Biotechnol Biofuels.

[12] Choix FJ, de Bashan LE, Bashan Y (2021). Enhanced accumulation of starch and total carbohydrates in alginate-immobilized *Chlorella * spp. induced by *Azospirillum brasilense*: II Heterotrophic conditions. Enzyme Microb Technol.

[13] Manhaeghe D, Blomme T, Van Hulle SWH, Rousseau DPL (2020). Experimental assessment and mathematical modelling of the growth of *Chlorella vulgaris* under photoautotrophic, heterotrophic and mixotrophic conditions. Water Res.

[14] Canelli G, Murciano Martínez P, Maude Hauser B, Kuster I, Rohfritsch Z, Dionisi F, Bolten CJ, Neutsch L, Mathys A (2021). Tailored enzymatic treatment of *Chlorella vulgaris* cell wall leads to effective disruption while preserving oxidative stability. Lwt Food Sci Technol.

[15] Morales-Sanchez D, Martinez-Rodriguez OA, Kyndt J, Martinez A (2015). Heterotrophic growth of microalgae: metabolic aspects. World J Microbiol Biotechnol.

[16] Tan XB, Zhao XC, Yang LB (2019). Strategies for enhanced biomass and lipid production by *Chlorella pyrenoidosa* culture in starch processing wastewater. J Cleaner Prod.

[17] Pleissner D, Lau KY, Ki Lin CS (2017). Utilization of food waste in continuous flow cultures of the heterotrophic microalga *Chlorella pyrenoidosa* for saturated and unsaturated fatty acids production. J Cleaner Prod.

[18] Cai Y, Liu Y, Liu T, Gao K, Zhang Q, Cao L, Wang Y, Wu X, Zheng H, Peng H, Ruan R (2020). Heterotrophic cultivation of *Chlorella vulgaris* using broken rice hydrolysate as carbon source for biomass and pigment production. Bioresour Technol.

[19] Xiao H, Yang F, Lin Q, Zhang Q, Zhang L, Sun S, Han W, Liu GQ (2020). Preparation and characterization of broken-rice starch nanoparticles with different sizes. Int J Biol Macromol.

[20] Myburgh MW, Cripwell RA, Favaro L, van Zyl WH (2019). Application of industrial amylolytic yeast strains for the production of bioethanol from broken rice. Bioresour Technol.

[21] Kumar Shetty AV, Dave N, Murugesan G, Pai S, Pugazhendhi A, Varadavenkatesan T, Vinayagam R, Selvaraj R (2021). Production and extraction of red pigment by solid-state fermentation of broken rice using *Monascus sanguineus* NFCCI 2453. Biocatal Agric Biotechnol.

[22] Xu E, Wu Z, Chen J, Tian J, Cheng H, Li D, Jiao A, Ye X, Liu D, Jin Z (2020). Calcium—lactate-induced enzymatic hydrolysis of extruded broken rice starch to improve Chinese rice wine fermentation and antioxidant capacity. Lwt Food Sci Technol.

[23] Maranon E, Cermeno P, Lopez-Sandoval DC, Rodriguez-Ramos T, Sobrino C, Huete-Ortega M, Blanco JM, Rodriguez J (2013). Unimodal size scaling of phytoplankton growth and the size dependence of nutrient uptake and use. Ecol Lett.

[24] Stolte W, Riegman R (1995). Effect of phytoplankton cell size on transientstate nitrate and ammonium uptake kinetics. Microbiology.

[25] Nelson DL, Cox MM. InLehninger Principles of Biochemistry. W.H. Freeman & Company 2000.

[26] Xie T, Xia Y, Zeng Y, Li X, Zhang Y (2017). Nitrate concentration-shift cultivation to enhance protein content of heterotrophic microalga *Chlorella vulgaris*: Over-compensation strategy. Bioresour Technol.

[27] Burrieza HP, Rizzo AJ, Moura Vale E, Silveira V, Maldonado S (2019). Shotgun proteomic analysis of quinoa seeds reveals novel lysine-rich seed storage globulins. Food Chem.

[28] Ramírez-Jiménez A, García-Villanova B, Guerra-Hernández E (2004). Effect of storage conditions and inclusion of milk on available lysine in infant cereals. Food Chem.

[29] Sibian MS, Saxena DC, Riar CS (2017). Effect of germination on chemical, functional and nutritional characteristics of wheat, brown rice and triticale: a comparative study. J Sci Food Agric.

[30] Mir NA, Riar CS, Singh S (2019). Effect of pH and holding time on the characteristics of protein isolates from *Chenopodium* seeds and study of their amino acid profile and scoring. Food Chem.

[31] Pietrysiak E, Smith DM, Smith BM, Ganjyal GM (2018). Enhanced functionality of pea-rice protein isolate blends through direct steam injection processing. Food Chem.

[32] Zhou T, Cao L, Zhang Q, Liu Y, Xiang S, Liu T, Ruan R (2021). Effect of chlortetracycline on the growth and intracellular components of *Spirulina platensis* and its biodegradation pathway. J Hazard Mater.

[33] Wang SK, Wang X, Tao HH, Sun XS, Tian YT (2018). Heterotrophic culture of *Chlorella pyrenoidosa* using sucrose as the sole carbon source by co-culture with immobilized yeast. Bioresour Technol.

[34] Gu Z, Liu Y, Zou G, Zhang Q, Lu R, Yan H, Cao L, Liu T, Ruan R (2021). Enhancement of nutrients removal and biomass accumulation of *Chlorella vulgaris* in pig manure anaerobic digestate effluent by the pretreatment of indigenous bacteria. Bioresour Technol.

[35] Song M, Pei H, Hu W, Ma G (2013). Evaluation of the potential of 10 microalgal strains for biodiesel production. Bioresour Technol.

[36] Dziekonska U, Kalinowska H (2015). Utilisation of sugar beet bagasse for the biosynthesis of yeast SCP. J Food Eng.

[37] Zhou P, Zhang L, Ding H, Gao X, Chen Y, Li D (2022). Optimization of culture conditions of screened *Galactomyces candidum* for the production of single cell protein from biogas slurry. Electron J Biotechnol.

